# The Impact of COVID-19 on Mortality in Spain: Monitoring Excess Mortality (MoMo) and the Surveillance of Confirmed COVID-19 Deaths

**DOI:** 10.3390/v13122423

**Published:** 2021-12-03

**Authors:** Inmaculada León-Gómez, Clara Mazagatos, Concepción Delgado-Sanz, Luz Frías, Lorena Vega-Piris, Ayelén Rojas-Benedicto, Amparo Larrauri

**Affiliations:** 1 National Centre of Epidemiology, Institute of Health Carlos III, 28029 Madrid, Spain; ileon@isciii.es (I.L.-G.); cmazagatos@isciii.es (C.M.); cdelgados@isciii.es (C.D.-S.); lorena.vega@isciii.es (L.V.-P.); ayelen.rojas@externos.isciii.es (A.R.-B.); 2 Consortium for Biomedical Research in Epidemiology and Public Health (CIBERESP), Institute of Health Carlos III, 28029 Madrid, Spain; 3 CIRCITER S.L., 28005 Madrid, Spain; luzfrias@gmail.com

**Keywords:** excess mortality, COVID-19, surveillance

## Abstract

Measuring mortality has been a challenge during the COVID-19 pandemic. Here, we compared the results from the Spanish daily mortality surveillance system (MoMo) of excess mortality estimates, using a time series analysis, with those obtained for the confirmed COVID-19 deaths reported to the National Epidemiological Surveillance Network (RENAVE). The excess mortality estimated at the beginning of March 2020 was much greater than what has been observed in previous years, and clustered in a very short time. The cumulated excess mortality increased with age. In the first epidemic wave, the excess mortality estimated by MoMo was 1.5 times higher than the confirmed COVID-19 deaths reported to RENAVE, but both estimates were similar in the following pandemic waves. Estimated excess mortality and confirmed COVID-19 mortality rates were geographically distributed in a very heterogeneous way. The greatest increase in mortality that has taken place in Spain in recent years was detected early by MoMo, coinciding with the spread of the COVID-19 pandemic. MoMo is able to identify risk situations for public health in a timely manner, relying on mortality in general as an indirect indicator of various important public health problems.

## 1. Introduction

The emergence of the coronavirus disease 2019 (COVID-19) pandemic was followed by a period of excess mortality in Europe and the UK, beginning in March until mid-May 2020 [1]. In Italy, the first European country affected by the COVID-19 outbreak, an estimated 47,490 excess deaths were observed between February and May 2020, most occurring in northern regions that suffered the greatest impact [2]. Portugal reported an excess mortality over 3 times higher than the official COVID-19 deaths between March and April 2020 [3] and England experienced the highest excess all-cause mortality since influenza season 2015/16 [4]. The first COVID-19 wave was also followed by a remarkable excess all-cause mortality in the United States [5,6].

Measuring the real impact of the COVID-19 pandemic on population mortality represents a great challenge, and it is known that the number of reported confirmed COVID-19 deaths in most countries greatly underestimates the total COVID-19-related mortality, particularly during the first months of the pandemic when severe acute respiratory syndrome coronavirus 2 (SARS-CoV-2) testing capacity was still limited [5]. During the first epidemic wave, all deaths occurring outside of the healthcare system, without ever being tested for SARS-CoV-2 infection, were not counted as COVID-19 deaths. Furthermore, deaths arising from untreated underlying health conditions due to limited access to the healthcare system, or those related with the indirect health effect or socioeconomic impact of the pandemic, such as unemployment or social isolation, are difficult to estimate in a timely manner and are frequently not recorded as part of the official COVID-19 mortality figures, even though they are a direct consequence of the pandemic’s impact on society and the pressure on the healthcare system [7]. 

The Spanish daily mortality surveillance system (MoMo) model and the information form EuroMOMO have been used for several years to monitor excess mortality in Spain, and it provides a valuable tool to better understand the mortality burden of the COVID-19 pandemic in the country. Additionally, it is essential to take into account the geographical distribution of the disease, as some regions were much more affected than others. As observed in other countries [2,8], impact on mortality can be very different between regions and national estimates can hide these differences.

We aimed to analyze COVID-19 mortality in Spain, describing several periods of excess mortality observed during the COVID-19 pandemic and providing estimates by age group and Spanish region and province. We compared the MoMo all-cause excess mortality during the COVID-19 pandemic with the confirmed COVID-19 deaths reported to the national surveillance system during the same periods, and with the MoMo excess mortality observed in recent years in Spain.

## 2. Materials and Methods

### 2.1. Data Sources

MoMo used daily all-cause deaths from the National Institute of Statistics (INE), until 2019. From 1 January 2020 to the present, daily mortality data were obtained through 3999 computerized civil registries, corresponding to 93% of the Spanish population and including all provinces. 

COVID-19 mortality data were obtained from notifications of death in confirmed COVID-19 cases reported by Autonomous Communities, within the exhaustive national COVID-19 surveillance of the National Epidemiological Surveillance Network (RENAVE) through the Web platform SiViES (Spanish Surveillance System), which is managed by the National Center of Epidemiology. A COVID-19 death is a patient death with a confirmed diagnosis of COVID-19 (PCR or rapid Ag within the first 5 days from symptom onset) in with another cause of death has not proved.

EuroMOMO mortality data were obtained from the weekly aggregated data of MoMo.

### 2.2. Statistical Methods

Expected mortality was estimated using a historical seasonal variation (centered 7-day moving averages among 10 years) based on the observed mortality of the last 10 years described earlier [9] and a non-linear secular trend (we subtract the median for each year from each day’s data and we add the median for the last year used in the estimation (2019)). Mortality for the year 2020 was removed to avoid overestimations. 

Recent MoMo data include a reporting delay [10]. To solve this, MoMo includes a delay correction model running over the last 28 days of observed mortality data. This delay is modelled with a survival analysis dependent on the autonomous community, the weekday (there is a lower ratio of reporting during the weekends) and the period in the year (more delay during holidays).

To extrapolate MoMo excess mortality to the national population, we used the notification rates (ratio between the deaths obtained from civil registries and the last available consolidated mortality series from the INE). Extrapolated mortality is estimated with the quotient between the observed and estimated deaths resulting from the model and the notification rate of their corresponding geographic area. In order to check the possible error on the extrapolation, we have compared the extrapolated national daily data from civil registries over the complete data (INE series). As the extrapolation ratios are calculated over 2019 (the last available year in the INE series), we have applied these ratios over the 2018 observed mortality (so we do not use the same data to validate it) and compared it to the complete data (Figure S1).

The following figure (Figure S1) shows that the extrapolated and complete data are quite similar.

The expected mortality with a 99% confidence interval was compared with the observed daily mortality. Excess mortality was calculated as the difference between the observed mortality and the expected mortality, by sex, age groups (0–14, 15–44, 45–64, 65–74, 75–84 and +85 years), Autonomous Community and province.

We calculated 14 days of cumulated excess death rates and 14 days of cumulated COVID-19 death rates using direct standardization with population data from the INE for each year. The reference population used was the European standard population (proposed by EUROSTAT in 2013) [11]. 

Excess all-cause mortality estimates by MoMo and reported confirmed COVID-19 deaths were obtained for the first three COVID-19 pandemic waves. The first wave was established from 1 March (the date on which the COVID-19 pandemic started) to 21 June 2020 (the date on which the first wave and the state of alarm were finalized). The second wave was from 22 June to 6 December 2020, and the third wave was from 7 December 2020 to 14 March 2021. These dates were established according to the inflection points on the COVID-19 14-day cumulative incidence curves.

We compared excess mortality from MoMo and EuroMoMo from 2015 to 2020 in order to show the important all-cause mortality experienced in 2020.

The analysis was conducted in R 4.0. The packages used were: lubridate, dplyr, ISOweek, ggplot2, yaml, RMySQL, nlme, readstata13, foreign, raster, reshape2, RColorBrewer, maptools, sp, rgeos, rgdal, sf, data.table.

## 3. Results

**Figure 1:**
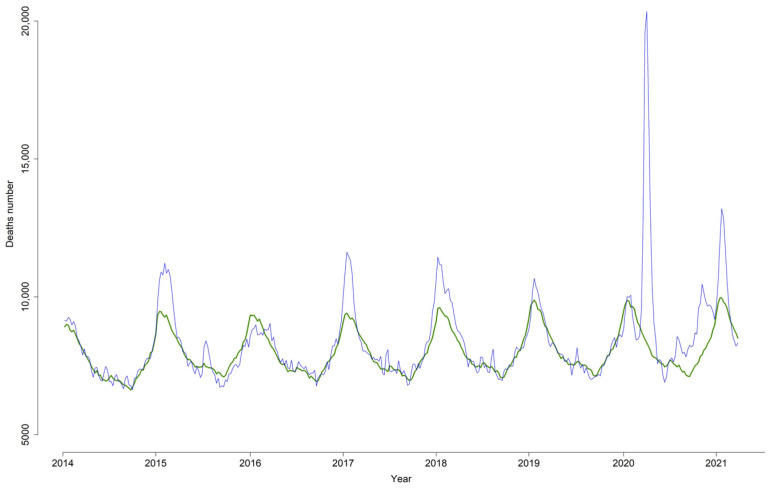
Weekly excess all-cause mortality (number of deaths). MoMo, Spain, 2014–2021.

Since 2014, MoMo has estimated excess mortality in several winters, corresponding to the influenza seasons 2014–2015, 2016–2017 and 2017–2018. Excess mortality was also observed during the summer of 2015 (Figure 1). Starting in March 2020, a large mortality excess was observed, concentrated in a very short time and higher than those observed in previous years. Other periods of excess mortality were identified in the last half of 2020 and the first half of 2021 (Figure 1).

**Figure 2:**
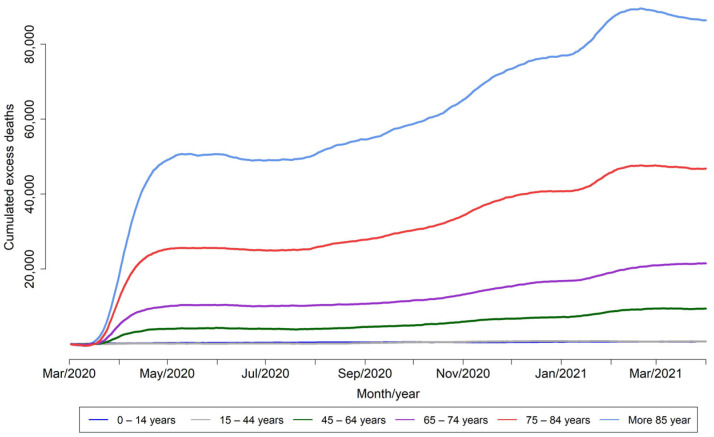
Daily cumulated excess all-cause mortality (number of deaths) by age group. MoMo, Spain, 2014–2021.

The cumulated excess mortality was greater as age increased. The highest cumulated mortality was observed in the 85 years and over age group, followed by the 75–84 age group (Figure 2).

**Figure 3:**
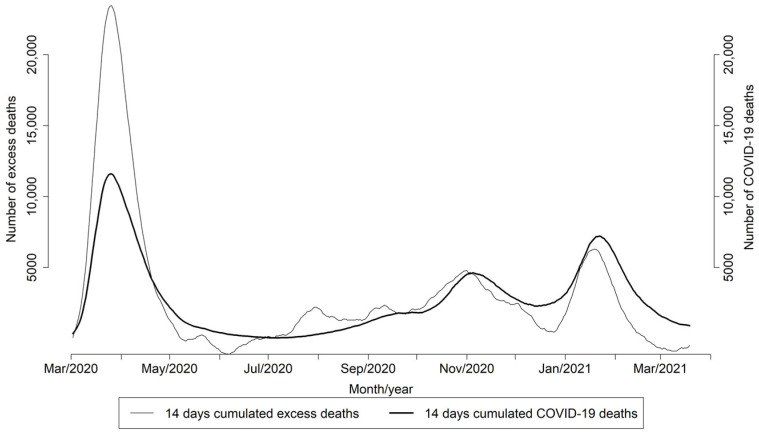
Daily excess all-cause estimated number of deaths by MoMo and COVID-19 deaths reported to RENAVE. Spain. March 2020–March 2021.

During the first epidemic wave, the excess mortality estimated by MoMo considerably exceeded the confirmed COVID-19 deaths reported to RENAVE, but this difference decreased in the following pandemic waves (Figure 3).

The ratio between the excess mortality estimated by MoMo and the confirmed COVID-19 deaths was 1.5 and 1.2 in the first and second pandemic waves, respectively, and higher than 1 for the age group 65 years and over, with the exception of the 15–44 age group in the second pandemic wave with a ratio of 2.5. In the third wave, the ratio was less than 1 for all age groups (Table 1).

MoMo excess mortality and the number of COVID-19 deaths reported to RENAVE were very heterogeneous between autonomous communities. The ratio between the mortality excess estimated by MoMo and the confirmed COVID-19 deaths was higher than 1 in all autonomous communities, except in Galicia, the Islas Canarias and Cantabria (Table 2). Among those with ratios higher than 1, the values ranged from 5.9 in Ceuta to 1.3 in Illes Baleares.

**Figure 4:**
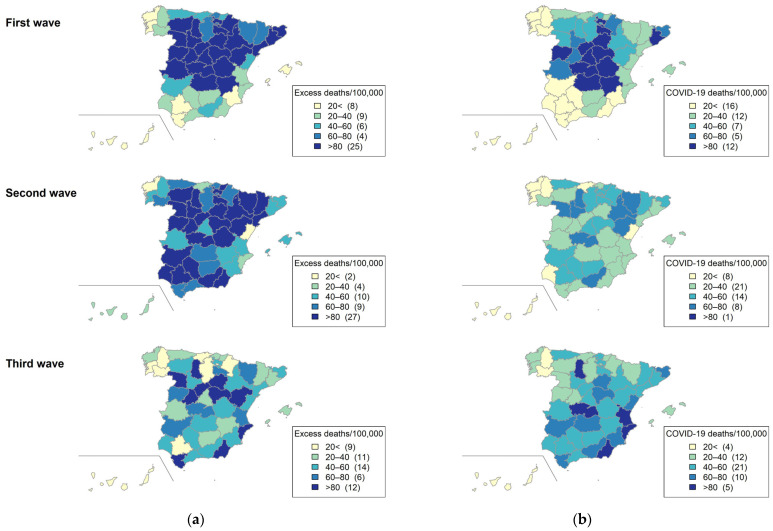
(**a**) Cumulated excess all-cause mortality MoMo age-standardized rates and (**b**) cumulated COVID-19 deaths age-standardized rates, by province. Spain, March 2020–March 2021.

In the first epidemic wave, excess mortality rates estimated by MoMo were higher in several autonomous communities and provinces in the center of Spain (Castilla y León, Castilla-La Mancha, Cáceres, Madrid, Huesca, Navarra, La Rioja, Álava, and Cataluña). In contrast, in the second wave, the highest excess mortality rates were observed in the south of Spain and several regions in the center and the north (Cuenca, Castilla y León, La Rioja, Álava, Guipúzcoa, Aragón, Lleida and Barcelona). In the third pandemic wave, the highest excess mortality rates were observed in Cádiz, Almería, Alacant, Ávila, Segovia, Soria, Guadalajara, Zamora Palencia and Teruel (Figure 4a).

In the first epidemic wave, confirmed COVID-19 mortality rates notified to RENAVE were higher in several provinces at the center and northeast of Spain (Ciudad Real, Toledo, Albacete, Cuenca, Madrid, Salamanca, Segovia, Guadalajara, Soria, La Rioja, Ávila and Barcelona). In the second epidemic wave, COVID-19 mortality rates were lower in all Spanish provinces. In the third epidemic wave, COVID-19 mortality rates were higher in several provinces at the center and east of Spain: Toledo, Palencia, València, Alacant y Almería (Figure 4b).

MoMo excess mortality and EuroMOMO excess mortality during the years 2015 to 2020 can be observed in Table 3. The highest excess detected by both systems occurred in 2020.

## 4. Discussion

Between March and May 2020, MoMo estimated an unprecedented all-cause excess mortality in Spain, coinciding with the first wave of the COVID-19 pandemic that was occurring in many countries around the world [1,4,5,8,12,13]. This all-cause excess mortality was also estimated by EuroMOMO in Spain and in other European countries: Belgium, England (UK), France, the Netherlands, Northern Ireland (UK), Portugal, Scotland (UK), Sweden, Switzerland and Wales (UK) [1]. June 2020 onwards, an increase in excess mortality was estimated, but it was not gathered in a short time. The age group with the greatest impact in terms of mortality was the older age group—those older of 75 years of age—although a strong impact was also seen in the 45–74 years age group.

As seen throughout 2020 in many other countries [14], our results show that all-cause excess mortality estimated by MoMo during the first epidemic wave in Spain was considerably higher than the confirmed COVID-19 deaths reported to RENAVE in the same period. In contrast, both estimates were very similar in the following two pandemic waves. This difference can be explained, on the one hand, by limited SARS-CoV-2 testing availability and diagnostic capacity during the first wave, causing underreporting of confirmed COVID-19 deaths to RENAVE during the first months of the pandemic [15], before an update in the national COVID-19 surveillance and control strategy was implemented in May 2020 [16]. In fact, the Spanish Ministry of Health along with the regional health departments introduced a global monitoring tool for monitoring COVID-19 tests carried out by public and private laboratories. The increase in testing capacity between the first and the third wave was 5.6 times [17]. Thus, a clear increase in testing capacity was reached after the first wave that helped to avoid underreporting of COVID-19 to RENAVE. Underreporting of confirmed mortality due to strained health system capacity to test and diagnose COVID-19 is common in many countries [14], and is also an issue when addressing seasonal influenza mortality. The total burden of influenza mortality is generally greater than confirmed influenza deaths [18], and instead of counting laboratory-confirmed deaths, influenza mortality is usually estimated. A standard approach for mortality estimations is to calculate the excess all-cause mortality, measured as the difference between the observed and expected all-cause deaths for a certain period of time. Excess mortality models have been widely used to estimate mortality during influenza seasons [9,19] and our results here show that the impact of COVID-19 on mortality during the first wave in Spain far exceeded the excess mortality observed during influenza seasons in previous years.

On the other hand, the Institute of Health Carlos III’s MoMo estimates in the first wave may also capture mortality “indirectly” related to COVID-19—that is, deaths from other non-COVID-19 causes but related to the pandemic situation—with more impact in the first wave compared to second and third waves: strict lockdown, social isolation, difficulty of access to medical care and the health system, among others, as was previously observed in other countries [8,13,20].

Despite the fact that COVID-19 was still present in our country, the MoMo excess mortality estimates in the second and third pandemic waves were lower compared to the first. Several factors may contribute to this general decrease in mortality observed throughout the pandemic. A “harvest” effect produced after a significant excess of deaths among elderly people whose health was already compromised [21], an improvement in the control of the COVID-19 epidemic in health centers or a better knowledge of the disease by professionals, with improved early health care and treatment against COVID-19, may have contributed to reducing the impact on mortality. In addition, COVID-19 vaccination started on 27 December 2020 in Spain and, therefore, it is also possible that impact of the vaccine might contribute to a reduction in COVID-19 mortality during the third wave (7 December 2020 to 14 March 2021), restricted to those groups where the vaccine was firstly prioritized, home residents, health care workers and then, the elderly. As vaccination coverage increased, the COVID-19 vaccine’s impact on the incidence and mortality of the population was more evident.

The age-adjusted mortality rates, both all-cause and confirmed with COVID-19, had a very different distribution among the different provinces of Spain in the different epidemic waves, as observed in other countries [2,22]. Different characteristics of Spanish provinces in terms of population density, age and mobility, as well as varying policies and control measures implemented by authorities at the local level, may explain these differences.

In summary, MoMo constitutes a valuable real-time surveillance tool that is capable of identifying situations of potential risk to public health in a timely manner. MoMo does not include meteorological variables or cause of death, so it does not allow causality to be established in the analysis of excess mortality. However, despite its lack of specificity due to the use of all-cause deaths, it has an important added value for public health surveillance, serving as a mortality alert system in Spain both at the national and local level. Despite the inherent limitations of surveillance systems, our results show the importance of general mortality as an indirect indicator of various important public health problems, and demonstrate that the MoMo system is able to monitor mortality and timely detect potential risk situations for public health.

## 5. Conclusions

In conclusion, we can assert that MoMo detected one of the main significant increases in mortality that occurred in Spain in recent years, which coincided with the emergence of the COVID-19 pandemic. The MoMo system is able to provide all-cause excess mortality estimates by age and geographic region, and is a valuable tool as an early warning system for public health action. 

## Figures and Tables

**Table 1:** Excess all-cause mortality estimates by MoMo and COVID-19 deaths reported to RENAVE by wave, Spain. March 2020–March 2021.

Period	Age Group	MoMo Excess Deaths (N)	COVID-19 Deaths (N)	Ratio MoMo/COVID-19 Deaths
First wave: 10 March 2020 21 June 2020	0–14	127	205	0.6
	15–44	129	214	0.6
	45–64	2064	2280	0.9
	65–74	5283	4391	1.2
	75–84	12,711	9168	1.4
	Older–85	24,730	13,190	1.9
	all	44,583	29,628	1.5
Second wave: 22 June 2020 6 December 2020	0–14	133	204	0.7
	15–44	362	147	2.5
	45–64	1336	1528	0.9
	65–74	3074	2451	1.3
	75–84	7330	5797	1.3
	Older–85	12,693	9869	1.3
	all	24,373	20,109	1.2
Third wave: 7 December 2020 14 March 2021	0–14	37	174	0.2
	15–44	39	178	0.2
	45–64	1243	2116	0.6
	65–74	2821	3848	0.7
	75–84	3610	7806	0.5
	Older–85	6291	11,571	0.5
	all	14,040	25,740	0.5
Total period	all	82,996	75,477	1.1

**Table 2:** Excess all-cause mortality estimates by MoMo and COVID-19 deaths reported to RENAVE by autonomous communities, Spain. March 2020–March 2021.

Autonomous Communities	MoMo Excess Deaths (N)	COVID-19 Deaths (N)	Ratio MoMo/COVID-19 Deaths
Andalucía	20,828	9630	2.2
Aragón	7048	3437	2.1
Ppdo. de Asturias	4097	1951	2.1
Illes Balears	1091	829	1.3
Canarias	−667	741	−0.9
Cantabria	267	560	0.5
Castilla-La Mancha	15,105	5857	2.6
Castilla y León	16,173	6729	2.4
Cataluña	32,794	14,164	2.3
C. Valenciana	15,443	7303	2.1
Extremadura	4774	1777	2.7
Galicia	2063	2378	0.9
C. Madrid	41,887	15,139	2.8
Región de Murcia	2648	1590	1.7
C. Foral de Navarra	2106	1167	1.8
País Vasco	6308	4281	1.5
La Rioja	1483	762	1.9
Ceuta	665	112	5.9
Melilla	356	95	3.7

**Table 3:** Excess all-cause mortality estimates by MoMo and EuroMOMO per year (2015–2019).

Year	MoMo Excess Deaths (N)	EuroMOMO Excess Deaths (N)
2015	8793	34,411
2016	4431	17,289
2017	16,121	28,543
2018	13,792	28,422
2019	201	16,098
2020	68,860	81,821

## Data Availability

MoMo data are available at: https://momo.isciii.es/public/momo/dashboard/momo_dashboard.html#datos (accessed on 11 May 2021). RENAVE data are available at: https://cneCOVID.isciii.es/COVID19/#documentaci%C3%B3n-y-datos (accessed on 11 May 2021).

## Supplementary Materials

The following are available online at https://www.mdpi.com/article/10.3390/v13122423/s1, Figure S1: Comparison between complete data mortality (INE, year 2018) and extrapolated national daily data (civil registries: 2019). R scripts used in the analysis.
