# Supplementary figures and images for: The Impact of COVID-19 on Mortality in Spain: Monitoring Excess Mortality (MoMo) and the Surveillance of Confirmed COVID-19 Deaths
Source: Viruses. 2021 Dec 3;13(12):2423. doi: 10.3390/v13122423 (PMC8703729; doi:10.3390/v13122423)

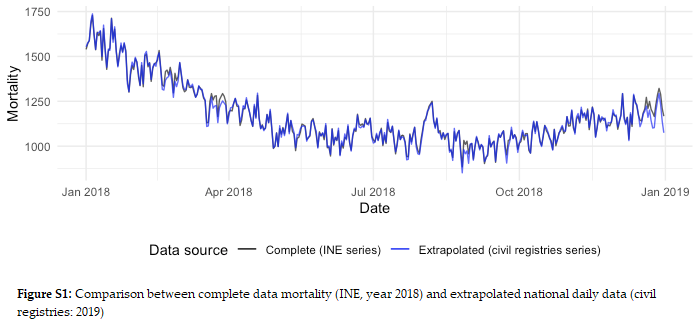

Supplement: Supplementary file 1 [file viruses-13-02423-s001.zip › FigureS1.png]
